# Checkpoint proteins come under scrutiny

**DOI:** 10.7554/eLife.01494

**Published:** 2013-10-08

**Authors:** Maria Mora-Santos, Jonathan BA Millar

**Affiliations:** 1 **Maria Mora-Santos** is in the Division of Biomedical Cell Biology, Warwick Medical School, University of Warwick, Coventry, United KingdomM.Mora-Santos@warwick.ac.uk; 2 **Jonathan BA Millar** is in the Division of Biomedical Cell Biology, Warwick Medical School, University of Warwick, Coventry, United KingdomJ.Millar@warwick.ac.uk

**Keywords:** spindle assembly checkpoint, kinetochore, Bub3, Bub1, Mad3, Knl1, *S. cerevisiae*

## Abstract

Details are emerging of the interactions between the kinetochore and various spindle checkpoint proteins that ensure that sister chromatids are equally divided between daughter cells during cell division.

**Related research article** Primorac I, Weir JR, Chiroli E, Gross F, Hoffmann I, van Gerwen S, Ciliberto A, Musacchio A. 2013. Bub3 reads phosphorylated MELT repeats to promote spindle assembly checkpoint signalling. *eLife* **2**:e01030. doi: 10.7554/eLife.01030

**Image** The checkpoint protein Bub3 (green) interacting with a MELT motif in the kinetochore

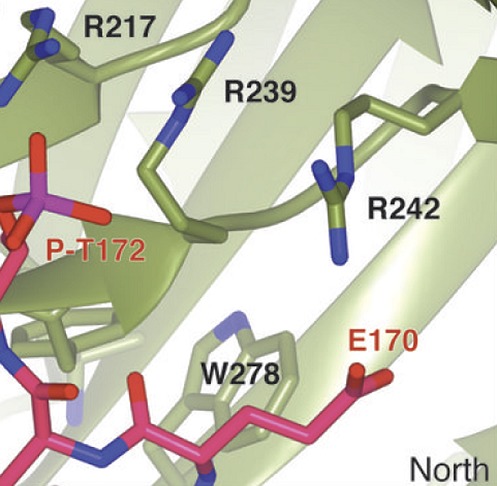

Before a cell can divide it must make a copy of all its chromosomes. After this has happened, each pair of identical chromosomes, which are known as sister chromatids, must be pulled apart, with one sister chromatid going into each daughter cell. Defective segregation of sister chromatids in human cells can lead to miscarriages, birth defects and diseases involving the proliferation of cells, such as cancer, so the process of chromosome segregation is rigorously controlled.

To ensure that sister chromatids are separated properly, a process known as chromosome bi-orientation must take place. This involves multi-protein structures called kinetochores, which form at the centre of each chromosome, another structure called the spindle, which has poles at opposite ends of the cell, and tubular polymers called microtubules that connect the kinetochores to the poles. Each sister chromatid has its own kinetochore, and chromosome bi-orientation involves one sister chromatid being connected to one pole, and the other sister chromatid being connected to the other pole. Once this has been achieved an enzyme called the APC/C is activated. This triggers removal of the glue that hold the sister chromatids together and the process of cell division can continue.

In addition to providing the physical linkage between the chromosomes and the microtubules, the kinetochore acts as the platform for a surveillance system called the spindle assembly checkpoint (SAC), which ensures that the APC/C is not activated until all chromosomes are correctly bi-oriented. The components of the SAC—which include various proteins (Mad1, Mad2, Mad3/BubR1 and Bub3) and kinases (Bub1 and Mps1)—are recruited to the kinetochores once the SAC has been activated. In particular, the recruitment of a Mad1-Mad2 complex is thought to herald the start of a series of events that ensures that APC/C is not activated (Sironi et al., 2002).

Only when all chromatids are correctly bi-oriented is the SAC switched off or ‘silenced’. However, it is still not clear what the SAC actually monitors. Several lines of evidence suggest that full occupancy of kinetochores by microtubules is required to silence the SAC (the attachment model), whereas other evidence suggests that intra-kinetochore stretch is also critically important (the tension model; Maresca and Salmon, 2009; Uchida et al., 2009). However, there is no detailed biochemical evidence to support either of these models. One of the reasons for this is that although the components of the spindle checkpoint were identified more than 20 years ago in yeast, their binding site (or sites) at kinetochores have, until recently, remained elusive. Now, in *eLife,* Andreas Musacchio and colleagues—including Ivana Primorac as first author—reveal the structural basis by which the Bub1 and Bub3 checkpoint proteins interact with the kinetochore (Primorac et al., 2013).

Important initial work by Mitsuhiro Yanagida and co-workers at Kyoto University pinpointed the KNL1 kinetochore protein as a likely receptor for the Bub1 and Mad3/BubR1 checkpoint proteins (Kiyomitsu et al., 2007). In particular they and others showed that the N-terminus of human KNL1 contains two KI motifs that bind Bub1 and Mad3/BubR1 (Figure 1). Crystal structures of these interactions have been reported (Bolanos-Garcia et al., 2012; Krenn et al., 2012).

**Figure 1.**
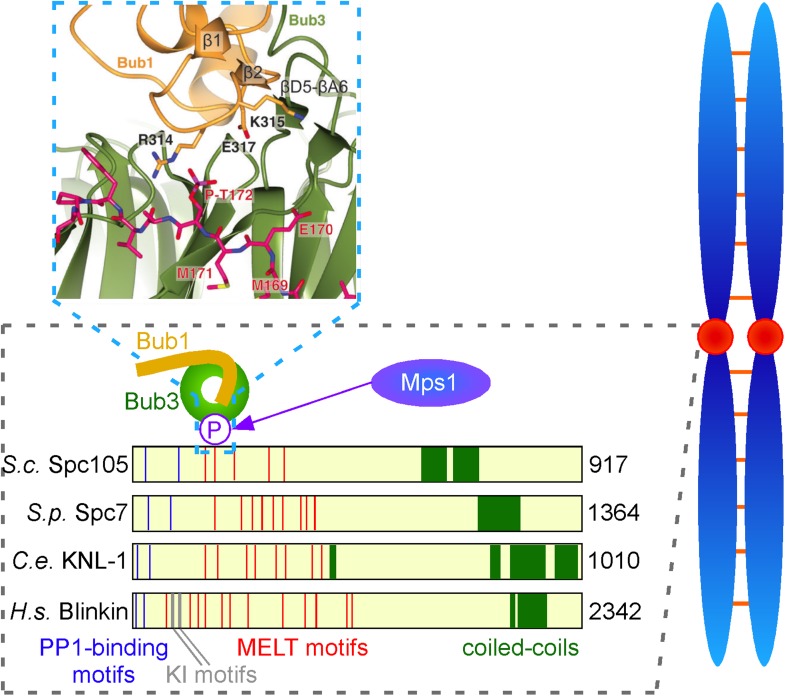
Interactions between checkpoint proteins the kinetochore. Before pairs of sister chromatids (shown in blue on the right) can be pulled apart during cell division, structures called spindle assembly checkpoints (SACs) form on the kinetochores (red circles) of each sister chromatid. The domain architecture of an important kinetochore protein called KNL1 is shown for four species, together with the name of the protein in that species and the number of amino acids it contains: *S. cerevisiae* (budding yeast, top); *S. pombe* (fission yeast); *C. elegans* (worm); Human (bottom). In experiments on budding yeast Primorac et al. have shown that the checkpoint protein Bub3 (green) binds to MELT motifs (red) that have been phosphorylated (P) by the enzyme Mps1, and that the checkpoint protein Bub1 (gold) then binds to Bub3 (and lies in almost the same plane as Bub3). A portion of the crystal structure displaying the interaction between Bub3, Bub1 and the phosphorylated MELT peptide (magenta) is also shown. The human version of KNL1 is the only version to have KI motifs (grey, see text); PP1-binding sites (blue) and coiled-coil kinetochore-binding domains (dark green) are also shown.

However, subsequent studies by the Musacchio lab showed these KI motifs are not essential for the association of Bub1 and Mad3/BubR1 to kinetochores (Krenn et al., 2012). Moreover, these motifs are absent from the homologues of KNL1 in yeast, so they may be less important in the recruitment of checkpoint proteins than was previous thought. More recently, several other groups, working principally with yeast, found that the KNL1 family of proteins contain a variable number of so-called MELT motifs (where M, E, L and T are all amino acids), and that these motifs, when phosphorylated by a kinase called Mps1, provide a binding site for the Bub3 and Bub1 proteins (London et al., 2012; Shepperd et al., 2012; Yamagishi et al., 2012).

Now, Musacchio and colleagues—who are based at the Max Planck Institute of Molecular Physiology in Dortmund, the IFOM laboratory in Milan and the University of Duisburg-Essen—show that a phosphorylated MELT peptide derived from budding yeast interacts with two ‘blades’ of the **β**-propeller in Bub3 (Figure 1). They go on to show that mutation of two basic residues in Bub3, which co-ordinate the phosphorylated threonine (T) residue of the MELT peptide, abolishes the interaction between the Bub3-Bub1 complex and the kinetochore, and therefore compromises checkpoint signalling. By demonstrating that Bub3 is the critical element that tethers Bub1 and Mad3/BubR1 to the kinetochore, Primorac et al. confirm an idea first put forward by Steven Taylor and colleagues over a decade ago (Taylor et al., 1998). Notably, however, the crystal structure suggests additional residues in the N-terminus of Bub1 contribute to this interaction (Figure 1). Although Bub3 also binds Mad3/BubR1, these additional stabilising residues are absent in the Mad3/BubR1 protein, suggesting that Mad3/BubR1 binds indirectly to KNL1 through an interaction with Bub1, rather than directly to the phosphorylated MELT motifs of KNL1.

Understanding the mechanical and biochemical basis of SAC signalling is one of the most challenging problems in cell biology. Although Primorac et al. establish the structural basis for the interaction of the Bub3-Bub1 complex with the kinetochore, many issues remain unresolved. First, it is not clear whether the MELT motifs are dephosphorylated once the spindle checkpoint is silenced, or which phosphatase catalyses this reaction, or whether dephosphorylation of these residues is important for the silencing process.

Second, structural data suggest that each MELT motif has the potential to bind one Bub3-Bub1 heterodimer. This raises the question as to why KNL1 and its homologues contain multiple MELT motifs (Figure 1)? One exciting possibility is that the arrays of MELT motifs in KNL1 act as a quantitative sensor of intra-kinetochore stretch. If this is the case, are unattached kinetochores monitored by the same, or a different, mechanism?

Third, it is not clear what role the interaction of Bub3-Bub1 with KNL1 plays in spindle checkpoint signalling in other organisms. For instance, the Bub3 protein is required for spindle checkpoint silencing in fission yeast, but activation can happen without it (Vanoosthuyse et al., 2009). Lastly, we still know very little about the interactions between other spindle checkpoint proteins—notably Mps1, Mad1 and Mad2—and the kinetochore.

Although the process of mitosis has been studied by scientists for over 100 years, the work of Primorac et al. suggests we can, nevertheless, be confident that full enlightenment will eventually emerge through the combined application of sophisticated molecular genetics, quantitative high-resolution live-cell imaging in various model systems, structural analysis of complexes and interaction surfaces, and biophysical analysis of the reconstituted system. But we may have to be patient for a little while longer.
